# Clinical presentation and outcome of Tuberculosis in Human Immunodeficiency Virus infected children on anti-retroviral therapy

**DOI:** 10.1186/1471-2431-8-1

**Published:** 2008-01-11

**Authors:** Elisabetta Walters, Mark F Cotton, Helena Rabie, H Simon Schaaf, Lourens O Walters, Ben J Marais

**Affiliations:** 1 Department of Pediatrics and Child Health, Faculty of Health Sciences, Stellenbosch University, Cape Town, South Africa; 2 Statistical analyst, Medscheme Health Risk Solutions, Cape Town, South Africa

## Abstract

**Background:**

The tuberculosis (TB) and human immunodeficiency virus (HIV) epidemics are poorly controlled in sub-Saharan Africa, where highly active antiretroviral treatment (HAART) has become more freely available. Little is known about the clinical presentation and outcome of TB in HIV-infected children on HAART.

**Methods:**

We performed a comprehensive file review of all children who commenced HAART at Tygerberg Children's Hospital from January 2003 through December 2005.

**Results:**

Data from 290 children were analyzed; 137 TB episodes were recorded in 136 children; 116 episodes occurred before and 21 after HAART initiation; 10 episodes were probably related to immune reconstitution inflammatory syndrome (IRIS). The number of TB cases per 100 patient years were 53.3 during the 9 months prior to HAART initiation, and 6.4 during post HAART follow-up [odds ratio (OR) 16.6; 95% confidence interval (CI) 12.5–22.4]. A positive outcome was achieved in 97/137 (71%) episodes, 6 (4%) cases experienced no improvement, 16 (12%) died and the outcome could not be established in 18 (13%). Mortality was less in children on HAART (1/21; 4.8%) compared to those not on HAART (15/116; 12.9%).

**Conclusion:**

We recorded an extremely high incidence of TB among HIV-infected children, especially prior to HAART initiation. Starting HAART at an earlier stage is likely to reduce morbidity and mortality related to TB, particularly in TB-endemic areas. Management frequently deviated from standard guidelines, but outcomes in general were good.

## Background

The tuberculosis (TB) epidemic is poorly controlled in sub-Saharan Africa; the region that reports the highest TB incidence rates and highest prevalence of human immunodeficiency virus (HIV) infection [1,2]. Prevention of mother to child transmission (PMTCT) programs are poorly established in many countries and in many instances fail to use potent antiretroviral regimens, resulting in huge numbers of HIV-infected children.

Childhood TB contributes significantly to the global TB case load (15–20% of cases), [3-5] especially in Africa where TB has been identified as a major respiratory cause of death in children [6]. The high disease burden results from ongoing TB transmission (poor epidemic control) and increased vulnerability as a result of HIV-induced immune compromise. Compared to HIV-uninfected children, HIV-infected children demonstrate greater morbidity and mortality from TB [5,7-11], as well as increased risk of rapid disease progression [12], unsatisfactory treatment response [7,8,13-15], and TB recurrence [13]. In resource-limited settings, mortality rates among HIV-infected children diagnosed with TB range from 20–35% [5,8,9,13-15], but the TB outcome in the era of highly active antiretroviral therapy (HAART) is less well documented.

With recent international efforts, HAART has become more widely available. The South African government supported the roll-out of HAART since 2004; all children with severe immune suppression and/or clinical stage 3 or 4 disease are eligible. HAART reduces the HIV viral load, which allows CD4 T-cell repletion and assists with the restoration of immunological function. Although complete functional immune reconstitution is rarely achieved, the beneficial effect of HAART in HIV-infected adults [16-19] and children [20-23] is well documented. In HIV-infected adults, HAART reduces the incidence of TB and the risk of TB recurrence [18,24,25], it also improves survival rates among TB patients [25-27]. A recent retrospective study from South Africa demonstrated a three-fold reduction in the incidence of TB in children on HAART compared to those not on HAART [28]. Restoration of specific antimycobacterial immune responses has been documented in children receiving HAART [29]; two recent case series described immune reconstitution inflammatory syndrome (IRIS)-related phenomena in children [30,31], but the prevalence and presentation of these phenomena require better description.

TB is regarded as a World Health Organization (WHO) HIV stage 3 (intra-thoracic) or 4 (extra-thoracic) condition. However, the best time for the introduction of HAART following a TB diagnosis remains uncertain; WHO recommends introducing HAART at different time points, starting earlier in the more immunocompromised children or where response to TB treatment is poor [32]. Early introduction of HAART carries a higher risk of IRIS-related phenomena, which may be misinterpreted as poor treatment response, drug-related adverse events or TB/HIV disease progression [30,33,34]; there is limited data in children to guide clinical decision making.

WHO recommends standard TB treatment, irrespective of the child's HIV status [22]. However, some uncertainty about the optimal treatment duration remains, due to reports of increased TB recurrence in HIV-infected children not on HAART who received standard TB treatment [6,7,23]. More information is required regarding TB recurrence in HIV-infected children and the influence of HAART. There are also concerns regarding common toxicities, drug interactions and treatment adherence if TB therapy and HAART are given concurrently. The aims of this study were to describe the clinical presentation and outcome of all children on HAART, in whom TB was diagnosed either during pre-HAART screening or during post-HAART follow-up.

## Methods

We conducted a retrospective descriptive study; data were collected by review of clinical files and chest radiographs. The files of all HIV-infected children (aged <13 years) who were initiated on HAART from 1 January 2003 through 31 December 2005 at Tygerberg Children's Hospital (TCH) were reviewed. We collected data on all TB episodes from the time of pre-HAART screening, defined as the 9 months prior to HAART initiation, until the end of the study period. Each TB episode was studied from treatment initiation until completion (where data was available), or death. Children who received single or dual antiretroviral therapy and children enrolled in TB prophylaxis trials were excluded.

### Data collection

Study subjects were identified from two sources; 1) the TCH pharmacy database, which record all children who received HAART since 2004, and 2) the HAART register kept at the HIV clinic that identifies children who accessed HAART prior to 2004. Clinical information was collected through a systematic search of clinical files kept at the HIV clinic and/or medical records. Demographic data, details of HIV clinical staging and HAART, as well as TB presentation and diagnosis were recorded for each episode. A chest radiograph (CXR) taken at the time of TB diagnosis was reviewed by a single childhood TB expert blinded to all clinical information. The outcome of each TB episode was assessed and categorized according to pre-defined outcomes.

### Definitions

HIV infection was defined as two positive HIV ELISA tests if over 18 months of age or a positive HIV RNA PCR test if below 18 months of age. HIV staging was defined according to the World Health Organization (WHO) clinical and revised immunological classification for HIV-associated immune-deficiency in infants and children (February 2006); TB was taken into account when allocating clinical categories. TB diagnosed more than 9 months prior to HAART initiation was recorded as previous TB. A TB episode included all TB diagnoses made within 9 months of HAART initiation or any time thereafter up to 31 December 2005. IRIS was defined as new mycobacterial disease and/or worsening of pre-HAART symptoms/signs recorded within the first 6 months after HAART initiation, in a child that demonstrated good immunological recovery and viral suppression.

The certainty of TB diagnosis was categorized as; 1) bacteriologically confirmed, where *M. tuberculosis *cultures were positive; 2) radiologically indicative, where cultures were negative or unavailable, but the CXR [or another radiographic investigation such as a computed tomography (CT)-scan] was considered indicative of TB; 3) clinically suspected, where cultures were negative or unavailable and radiographic evidence missing or not indicative of TB, but the child presented with a persistent cough for more than 2 weeks and/or fever for more than 1 week and/or recent failure to thrive, together with documented TB contact and/or a positive tuberculin skin test (TST, defined as an induration size ≥5 mm using the Mantoux test). Alternatively specific organ involvement, such as neck stiffness, a depressed level of consciousness, focal signs and cerebrospinal fluid findings indicative of TB meningitis. Positive treatment response provided additional supportive evidence. Not TB was the outcome if an alternative diagnosis was established (2 cases) and/or where critical review of the data provided insufficient evidence to support a diagnosis of TB (this occurred in 1 case).

Standard TB treatment comprised of isoniazid (INH), rifampicin (RMP) and pyrazinamide (PZA) for 2 months, followed by INH and RMP for 4 months; expanded regimens included ethambutol (EMB) and/or ethionamide (ETH) in addition. Alternative TB regimens included second line TB drugs such as ofloxacin, ciprofloxacin, amikacin, and/or cycloserine/terizidone. The outcome of each TB episode was categorized as; 1) cure, where there was good clinical response to treatment and follow-up cultures were negative in an episode of bacteriologically confirmed TB; 2) treatment completed and child well, where the response to treatment was good in an episode that did not have bacteriological confirmation, or where follow-up cultures were not available; 3) improvement, where some symptoms persisted but the child was assessed as clinically better than at TB diagnosis; 4) no improvement, where the original symptoms persisted or worsened; and 5) death, if child died before completing TB treatment. Outcomes 1–3 were grouped together as positive outcome. An adverse event was defined as any event resulting in modification of TB treatment or HAART either because of suspected drug toxicity or as a precautionary measure.

### Statistical analysis

Data were entered into a Microsoft SQL Server database. Statistical calculations were performed using SAS version 9.1. Descriptive analyses were performed and the Z test for independent proportions was used to calculate the difference between two sample proportions. Differences in median values were calculated by the Mann-Whitney-Wilcoxon test. A p-value of <0.05 was considered significant. The number of TB cases per 100 patient years was calculated using the formula; number of cases × 12/observation period in months × 100/number of patients observed. This calculation was performed in two groups of children; 1) those where TB was diagnosed in the 9 months prior to HAART initiation and 2) those where TB was diagnosed during the post-HAART follow up period (average 13.5 months).

Ethics approval was granted by Stellenbosch University's Committee for Human Research (project number N06/08/167).

## Results

**Figure 1:**
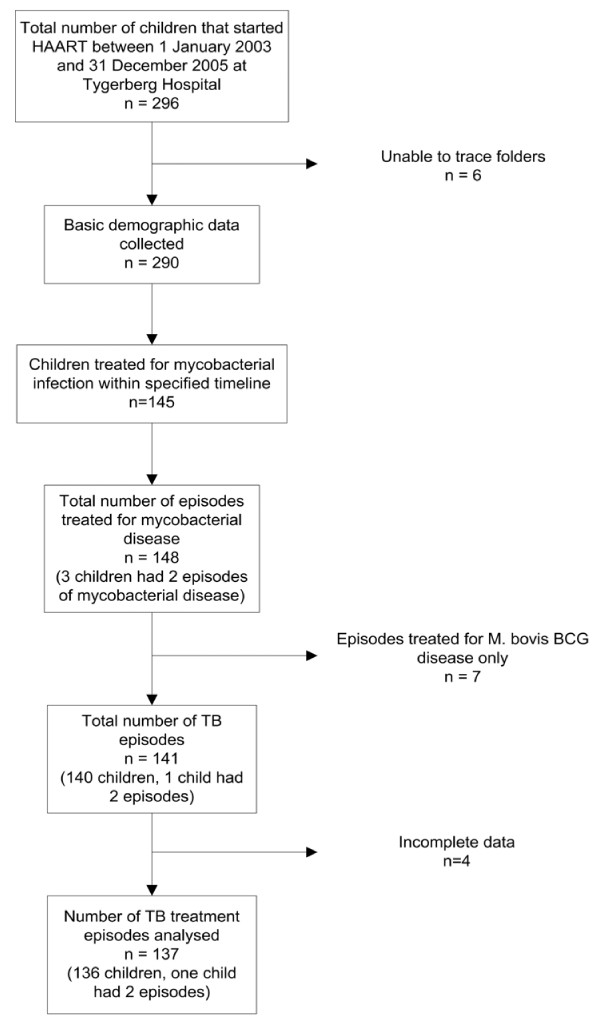
Schematic overview of the study population.

During the study period 296 children started HAART; 6 folders could not be located. Of the remaining 290 children 140 (48.3%) received TB treatment; 4 cases had incomplete data. The final group analyzed consisted of 136 children, of whom one had two TB episodes during the study period. Figure 1 provides a schematic overview of the study population. Table 1 reflects basic demographic data, HIV staging, weight and previous TB episodes recorded at HAART initiation in all 140 children treated for TB. There were significantly more children with severe immunosuppression among those diagnosed with TB compared to those without TB (WHO severe CD4 depletion 117/140 versus 94/150, p < 0.0001), and more children with TB were under their expected weight for age (<60% EWA 29/140 versus 14/150; 60–80% EWA 64/140 versus 46/150, p < 0.01 for both).

**Table 1:** Basic demographic data, HIV staging, weight categorization and previous TB episodes recorded at the time of HAART initiation, comparing those treated for TB to those not treated for TB

	**All children who received HAART**	**Treated for TB during study period**	**Not treated for TB during study period**	**p-value**
**Total number**	290 (100%)	140^#^‡ (100%)	150^## ^(100%)	
**Gender**				
**Male**	143 (49%)	67 (48%)	76 (51%)	0.43
**Age (months)**				
**Range/median**	1–160/23.5	2–140/19	1–160/33.5	0.08
WHO HIV staging*				
**CD4 depletion**				
**not significant**	22 (8%)	4 (3%)	18 (12%)	NA
**mild**	26 (9%)	9 (6%)	17 (11%)	0.14
**advanced**	31 (11%)	10 (7%)	21 (14%)	0.06
**severe**	211 (73%)	117 (84%)	94 (63%)	<0.001
**Clinical stage**				
**II**	15 (5%)	1 (1%)	14 (9%)	NA
**III**	128 (44%)	50 (36%)	78 (52%)	<0.01
**IV**	144 (50%)	89 (63%)	55 (37%)	<0.001
**Unknown**	3 (1%)	0 (0%)	3 (2%)	NA
**Weight categories**				
<60% EWA**	43 (15%)	29 (21%)	14 (9%)	<0.01
**60–80% EWA**	110 (38%)	64 (46%)	46 (31%)	<0.01
**80–100% EWA**	92 (32%)	34 (24%)	58 (39%)	<0.01
**>100% EWA**	25 (9%)	6 (4%)	19 (13%)	0.01
**Unknown**	20 (7%)	7 (5%)	13 (8%)	0.22
**Previous TB**				
**1 episode**	76 (26%)	19 (14%)	57 (38%)	<0.001
**≥2 episodes**	12 (4%)	7 (5%)	5 (3%)	NA

*WHO, World Health Organization immunological classification of HIV-associated immunodeficiency in infants and children, 2006

**EWA, expected weight for age

^# ^Includes 4 children subsequently excluded due to incomplete data

^## ^Includes 5 children with BCG disease only, who were excluded from further TB analysis

NA – not applicable, sample proportions too small for Z test

Table 2 reflects 137 TB episodes for which sufficient data could be obtained. A single child with two TB episodes, the first episode before and the second 5 weeks after HAART, is represented twice. In total, 116/137 (84.7%) TB episodes occurred prior to HAART initiation. Of these children 4 started TB treatment and HAART simultaneously, 80 were still on TB treatment when HAART was commenced and 22 first completed their TB treatment; the date of TB treatment completion was unknown in 10. The number of TB cases per 100 patient years, calculated for the different groups were; 1) 53.3/100 patient years during the 9 months prior to HAART initiation, and 2) 6.4/100 patient years during the period of post-HAART follow-up (average 13.5 months) [odds ratio (OR) 16.6; 95% confidence interval (CI) 12.5–22.4].

**Table 2:** All children diagnosed with TB during the study period, differentiating between those diagnosed before and after HAART initiation

	**Children treated for TB**	**TB diagnosed <9 months prior to HAART**	**TB diagnosed <6 months after HAART**	**TB diagnosed ≥6 months after HAART**
**Total number**^#^	137 (100%)	116 (100%)	14 (100%)	7 (100%)
**Gender: Male**	64 (47%)	54 (47%)	6 (43%)	4 (57%)
**Age (months):**				
**Range/median**	2–140/18	2–135/20	3–140/17	3–75/17
**HIV staging**				
WHO* CD4 depletion				
**not significant**	5 (4%)	3 (3%)	1 (7%)	0 (0%)
**mild**	9 (7%)	7 (6%)	0 (0%)	2 (29%)
**advanced**	10 (7%)	7 (6%)	2 (14%)	1 (14%)
**severe**	113 (82%)	98 (84%)	11 (79%)	4 (57%)
**WHO clinical stage**				
**II**	1 (1%)	0 (0%)	1 (7%)	0 (0%)
**III**	47 (34%)	40 (34%)	5 (36%)	2 (29%)
**IV**	89 (65%)	76 (66%)	8 (57%)	5 (71%)
**Weight categories**				
<60% EWA**	27 (20%)	24 (21%)	3 (21%)	0 (0%)
**60–80% EWA**	64 (47%)	51 (44%)	7 (50%)	6 (86%)
**80–100% EWA**	34 (25%)	31 (27%)	3 (21%)	0 (0%)
**>100% EWA**	6 (4%)	5 (4%)	1 (7%)	0 (0%)
**Unknown**	6 (4%)	5 (4%)	0 (0%)	1 (14%)
**Previous TB**				
**1 episode**	18 (13%)	12 (10%)	5 (36%)	1 (14%)
**≥2 episodes**	7 (5%)	4 (3%)	1 (7%)	2 (29%)

^#^4 folders with incomplete data excluded. 1 child with 2 TB episodes is represented twice.

*WHO, World Health Organization immunological classification of HIV-associated immunodeficiency in infants and children, 2006

**EWA, expected weight for age

### Clinical presentation

The presence or absence of a TB index case was recorded in 107/137 (78%) episodes; positive contact was identified in 53/107 (49.5%). In 29, the TB contact was an adult member of the household who had started TB treatment in the preceding 6 months. Sputum smear results were recorded in 10 cases; 2 were positive. In 116/137 (84.7%) episodes the child presented with at least one sign or symptom suggestive of TB; 2 or more symptoms were recorded in 70/137 (51.1%). The most common presenting symptoms were weight loss or failure to thrive (90; 65.7%), cough >2 weeks (50; 36.5%), and intermittent fever >1 week (43; 31.4%). Lethargy or irritability were recorded in 33 cases (3 had TB meningitis) and 14 had a history of recurrent or persistent chest infections.

Table 3 demonstrates the spectrum of mycobacterial disease recorded in the 136 children analyzed. Five children treated for TB had concurrent *Mycobacterium bovis *bacillus Calmette-Guérin (BCG) axillary adenitis. Two cases of NTM disease were initially treated for TB; 1 cultured *M. fortuitum *from 2 gastric aspirates, the other had a single positive gastric aspirate culture of an unspecified NTM. Among the 33 *M. tuberculosis *cases with extra-thoracic disease manifestations, the sites most commonly involved were abdomen (10; 30.3%), central nervous system (9; 27.3%), miliary disease (9; 27.3%), cervical/axillary lymph nodes (4; 12.1%), and pericardium (2; 6.1%). There were also 2 cases of probable congenital TB with hepatomegaly.

**Table 3:** Spectrum of mycobacterial disease recorded in HIV-infected children treated for TB

**Diagnosis**	**Number (%)**
***M. tuberculosis***	135 (98.5)
Intra-thoracic	102 (74.5)
Extra-thoracic	5 (4)
Intra- and extra-thoracic*	28 (20)
Concurrent *M. bovis *BCG**	5
Localized (axillary adenitis)	5
Distant or disseminated	0
**Non-tuberculous mycobacteria**	
Pulmonary^#^	2 (1.5)
**TOTAL**	137 (100%)

*9 cases of miliary TB are included in this group

**BCG: bacillus Calmette-Guérin

^#^Non-tuberculous mycobacteria cultured from gastric aspirates

Table 4 reflects the presenting features; CXR data and/or culture results were available in 107 (89.1%) cases, the remainder was classified as "clinically suspected TB". Bacteriologically confirmed TB was diagnosed pre-HAART in 40/116 (35%) and post-HAART in 6/21(29%) cases; the number of bacteriologically confirmed TB cases per 100 patient years were 18.3 pre-HAART and 1.8 post-HAART. Three cases were classified as "not TB". Of these, one child presented with weight loss, marasmus and liver dysfunction, but was ultimately diagnosed with candida septicaemia. There was no history of TB contact, 2 gastric aspirates yielded negative cultures and the CXR was normal. In the other 2 cases a NTM organism was cultured. There was no statistical difference in TST reactivity, contact history or proportion with suggestive symptoms among the diagnostic categories. TST results were positive in only 16/73 (22%) children with bacteriologically confirmed and/or radiologically indicated TB.

**Table 4:** Presenting features of TB episodes, differentiated according to level of diagnostic certainty

**TB diagnostic category**	**Total number (%)**	**TB contact**	Positive TST*	Suggestive symptoms**
**Bacteriologically confirmed**	46 (100)	16 (33)	12 (25)	37 (80)
**Radiologically indicated**	27 (100)	9 (33)	4 (15)	25 (93)
**Clinically suspected**	61 (100)	28 (46)	17 (29)	51 (82)
**Not TB**	3 (100)	0 (0)	0 (0)	3 (100)
**TOTAL**	137 (100)	53 (39)	33 (24)	116 (85)

*TST, tuberculin skin test

**Persistent cough for >2 weeks and/or fever for >1 week and/or recent failure to thrive and/or specific organ involvement suggestive of TB

Among children who started HAART before the end of TB therapy, few developed clinical exacerbation of disease after HAART initiation, apart from 2 who developed concurrent BCG axillary adenitis. Of the 14 children diagnosed with TB within 6 months of HAART initiation, 12 had severe and 2 advanced immune suppression. Ten children had excellent immunological and virological responses following HAART [mean CD4% before and after HAART (11.2% versus 28.2%); mean HIV viral load before and after HAART (log 6.1 versus log1.1)], while 3 showed poor response and one was lost to follow-up. Among the 10 children with vigorous immune reconstitution, those most likely to be true cases of IRIS, all episodes occurred between 2 weeks and 4 months after HAART initiation; 5 were bacteriologically confirmed cases. CXR's were not remarkable for extensive or cavitating disease.

### TB treatment

Of the 137 TB episodes analyzed, 91 (66%) were initially treated with the standard 3-drug regimen and 46 (34%) were treated with expanded or alternative regimens. Expanded regimens were given for cavitating or extensive intrathoracic disease (3), extra-thoracic or miliary disease (19), previous TB (10) and for concurrent *M. bovis *BCG disease (2). Alternative regimens were given for drug-resistant disease (3), pre-existing hepatitis (3), extensive intra-thoracic disease (1), and for concurrent *M. bovis *BCG disease (2); reason unclear (3). In 29 (21%) cases the initial TB regimen was subsequently changed; reasons for changing to a different regimen are reflected in Table 5.

**Table 5:** Reasons for changing the TB treatment regimen in episodes treated initially with standard or expanded regimens

Reason for regimen change	**Initial regimen**		**Total (%)**
	HRZ*	HRZ ± Eth ± E*	
Persistent culture-positive/progressive disease	10	2	12 (38)
Drug resistance	3	4	7 (22)
Adverse event^#^	3	6	9 (28)
*M. bovis *BCG/NTM**	3	1	4 (12)
Total (%)^##^	19 (59)	13 (41)	32 (100)

*H = isoniazid, R = rifampicin, Z = pyrazinamide, Eth = ethionamide, E = ethambutol,

**M. bovis BCG = *Mycobacterium bovis *Bacillus Calmette-Guérin disease, NTM = non-tuberculous mycobacteria disease

^#^In total there were 13 adverse events, only 9 appear in this table as the others did not necessitate a regimen change (2 started on alternative TB regimens, 1 stayed on the initial regimen, 1 had all treatment suspended)

^##^In total 32 regimen changes were recorded in 29 cases; 3 changed regimens twice

Adverse events were noted in 13/137 (9.5%) episodes: 8 had raised liver enzymes (>5 times the upper limit of normal), 4 had skin reactions and 1 developed anaemia and thrombocytopenia. Table 6 outlines the adverse events recorded, their likely causation and temporal relationship to TB treatment and HAART. Only 7/137 (5%) events were probably drug related: 5 cases of raised liver enzymes and 2 urticarial reactions. Drug related adverse events occurred in 3 children on concurrent HAART and TB treatment. TB treatment was suspended or modified in all cases with raised liver enzymes, except in one case where an elevated alanine transaminase level done routinely for HAART toxicity monitoring went unnoticed until the child's next visit a month later, by which time it had resolved.

**Table 6:** Adverse events recorded during TB treatment according to the type of event, likely cause and relation to TB treatment and/or HAART

**Type of adverse event**	**Likely cause**	**Relation to TB Rx**	**Relation to HAART**	**TB Rx regimen**	**TB Rx change**	**HAART outcome**	**Clinical outcome**
**Raised liver enzymes n = 8**							

Transaminitis (severe*)	Auto-immune haemolysis	6 weeks	No HAART	HRZE	Alternative Rx 3 months	N/A	Resolved
All enzymes (severe*)	Uncertain	5 months	1 month before TB Rx	HRZEO	Stopped	Suspended 2 months	Resolved
Raised ALT (severe*)	TB drugs	2 months	>2 months after TB Rx	HRZE	Unchanged	Unchanged	Resolved
All enzymes (moderate*)	TB drugs (PZA)	1 week	No HAART	HRZE	Alternative Rx 6 months	N/A	Resolved
Transaminitis (severe*)	TB drugs (PZA)	1 week	1 month before TB Rx	HRZE	Alternative Rx 3 months	Suspended 1 week	Resolved
Raised canalicular enzymes (severe*)	Uncertain	4 months	>2 months after TB Rx	HRZ	Alternative Rx 2 months	Unchanged	Resolved
All enzymes (mild*)	CMV disease	2 months	None	HRZEth	Alternative Rx 1 month	N/A	Died
Transaminitis (severe*)	Klebsiella sepsis	3 weeks	1 month after TB Rx	HRZE	Alternative Rx 1 day	Suspended	Died

**Skin reaction n = 4**							

**Urticarial**	PZA	1 day	None	HRZE	PZA stopped	N/A	Resolved
**Mild unspecified**	Uncertain	9 months	>2 months after TB	HZEEthAT	Unchanged	Unchanged	Resolved
**Urticarial**	Possibly PZA	1 day	None	HRZ	Alternative Rx 2 months	N/A	Resolved
**Urticarial**	Uncertain	2 months	<2 months after TB	HRZ	Alternative Rx 1 month	Suspended 1 month	Resolved

**Bone marrow suppression n = 1**							

**Anaemia and low platelets**	Sepsis	3 weeks	None	HRZEth	Suspended	N/A	Died

PZA/Z = pyrazinamide, H = isoniazid, R = rifampicin, E = ethambutol, Eth = ethionamide, O = ofloxacin, A = amikacin, T = terizidone, Strep = streptomycin, Cipro = ciprofloxacin, CMV = cytomegalovirus; N/A = not applicable

*Degree of enzyme elevation: mild = 2–5x normal value; moderate = 5–10x normal value; severe = >10x normal value

The outcome related to the duration of treatment is reflected in Table 7; the 3 children deemed not to have had TB were not included in this analysis. Of 134 TB episodes, 33 (25%) were treated for 6 months; 15 were treated for <6 months of whom 12 died and 3 (none with bacteriologically confirmed or radiologically certain TB) had a positive outcome. Thirty eight (28%) episodes were treated for 7–9 months; 9 had TB previously, 2 had concurrent BCG adenitis, 3 experienced initial deterioration and the remaining 24 were treated for longer due to their HIV status. Nineteen (14%) episodes were treated for 10–12 months; 4 had drug-resistant disease, 2 INH-monoresistant and 2 multidrug-resistant (MDR) TB; 1 received alternative treatment due to a drug-related adverse event; 4 had extensive intrathoracic disease and/or experienced disease progression on treatment; 1 developed concurrent BCG adenitis. It was uncertain why the remaining 9 children received prolonged treatment. Seventeen (13%) episodes received TB treatment for >12 months; 7 had confirmed MDR TB, 1 had pre-existing hepatitis that necessitated alternative therapy, 4 had persistent culture positive or progressive disease after 3 months of treatment, 4 experienced drug related adverse reactions, 1 developed concurrent BCG adenitis and another reported 2 previous episodes of TB.

**Table 7:** Outcome of TB episodes in relation to duration of TB treatment

**TB outcome**	**Duration of TB treatment**						**Total (%)**
		
	<6 months	6 months	7–9 months	10–12 months	>12 months	Unknown	
**Cure**	1	4	4	1	7	0	17 (12)
**Clinically well**	1	17	15	7	3	0	43 (32)
**Improved**	1	10	13	7	3	0	34 (27)
**Not improved**	0	0	3	2	0	1	6 (4)
**Death before end of TB treatment**	12	0	2	1	1	0	16 (12)
**LTF/TFO/Unknown**	0	2	1	1	3	11	18 (13)
**Total (%)**	15 (11)	33 (25)	38 (28)	19 (14)	17 (13)	12 (9)	134(100)

LTF = lost to follow-up; TFO = transferred out

### TB outcome

A positive outcome was achieved in 97/137 (71%) episodes: cure in 17 (12%), treatment completed and child well in 45 (33%), improved in 35 (26%). In 6 (4%) cases no improvement was documented; 16 (12%) children died before TB treatment was completed. Outcome could not be established in 18 (13%) episodes, where children were lost to follow up. All 6 episodes that failed to show improvement were diagnosed before HAART initiation; 1 had disseminated drug-susceptible TB meningitis, 1 had biopsy-proven lymphoid interstitial pneumonitis with extensive bronchiectasis, the remaining 4 were all severely immunocompromised and had significant co-morbidities. Positive outcomes were achieved in 80/116 (69%) TB episodes diagnosed pre-HAART, 11/14 (78%) episodes diagnosed <6 months and 6/7 (86%) episodes diagnosed ≥6 months post-HAART. HAART was introduced <2 months after the start of TB therapy in 30 children, all had advanced/severe HIV disease; the outcome was not documented in 4 cases. A good outcome was achieved in 17/26 (65%) and 9/26 (35%) had a poor outcome; 8 died and 1 did not improve.

Table 8 reflects demographic data, TB diagnosis and HIV staging at death of the 16 children who died before completing TB therapy. It also reflects the timing of death in relation to the initiation of TB treatment and HAART. Apart from 1 child who developed TB <3 months after starting HAART, 15/16 (93.8%) deaths occurred in children diagnosed with TB before HAART initiation. The child on HAART died in the third month of TB treatment from intestinal perforation secondary to colitis. The etiology was not confirmed, but might have been related to IRIS as the child showed good immunological recovery; CD4 count at HAART initiation 19% versus 27% at death. Mortality was reduced in those already on HAART (1/21; 4.8%) compared to children who started HAART after TB diagnosis [15/116; 12.9% – odds ratio (OR) 0.34 95% confidence interval (CI) 0.01–2.46].

**Table 8:** Summary of all children who died before completing TB therapy; demographic data, HIV staging, TB diagnosis and timing of death in relation to TB treatment and HAART initiation

	**Number (%)**
**Total deaths**	16 (100)
**Gender **(Male)	8 (50)
**Age **(months); Median (range)	11.5 (4–110)
WHO* CD4 depletion	
not significant	2 (13)
mild	0 (0)
advanced	1 (6)
severe	13 (81)
**WHO clinical stage**	
III	1 (6)
IV	15 (94)
**Disease manifestation**	
Intra-thoracic TB	8 (50)
Extra- and intra-thoracic TB	5 (31)
Culture-confirmed *M. tuberculosis*	7 (44)
MDR** TB	2 (13)
TB & BCG^# ^disease	1 (6)
NTM^##^	1 (6)
**Timing of death in relation to start of TB treatment**	
<2 months	6 (38)
≥2 months	10 (62)
**Timing of death in relation to HAART initiation**	
<2 weeks	5 (31)
2 weeks – 6 months	9 (56)
>6 months	2 (13)

*WHO, World Health Organization immunological classification for HIV-associated immunodeficiency in infants and children, 2006

**MDR – multi-drug resistant (resistant to at least INH and RMP)

^#^BCG – *M. bovis *Bacillus Calmette Guérin;

^##^NTM – non-tuberculous mycobacteria

Of the 15 children first diagnosed with TB that died, 8 (53%) started HAART within 2 months of commencing TB treatment. Six of the 8 deaths occurred early (within 3 weeks of HAART initiation); 5 were severely immunocompromised (2 died of pneumonia, 3 had chronic gastroenteritis with/without severe electrolyte disturbances and/or septicaemia) and the sixth child died as a result of co-existent congenital heart disease. All 6 were infants (<1 year of age). Two children died later in the course of TB treatment, 1 died 5 months into TB treatment and 3 months after HAART initiation from disseminated MDR TB, 1 died 3 months into TB treatment and 2 months after HAART from bacterial pneumonia.

The remaining 7 deaths occurred in children who commenced HAART ≥2 months after starting TB therapy. Four children died <6 months after HAART initiation; 1 child with chronic lung disease and culture-confirmed TB died 9 days later from bacterial pneumonia, 1 died 3 weeks later from intestinal perforation secondary to *Entamoeba enterocolitis *infection and septicaemia, 1 infant with miliary TB died 2 months later from "aspiration pneumonia", 1 child with pulmonary NTM disease died 1 month after HAART initiation from progressive pneumonia. Three children with culture-confirmed TB died ≥ 6 months after HAART initiation; 1 from bacterial pneumonia and septicaemia, 1 developed severe upper airway obstruction of uncertain etiology and 1 died from MDR TB meningitis.

Four deaths may have been partly attributable to IRIS; two cases, 1 with intestinal perforation and 1 with acute upper airway obstruction, showed good immune recovery at death compared to baseline, but occurred more than 5 months after HAART initiation. Two other deaths also demonstrated excellent immune recovery on HAART; one child with MDR TB developed multiple TB abscesses 3 weeks after HAART and died shortly thereafter, another child with MDR TB had TB meningitis died from acute hydrocephalus 5 months after HAART. However, it is impossible to establish with certainty whether IRIS was a contributory cause of death in these children.

## Discussion

During the study period, 48% of HIV-infected children received TB treatment. This confirms the high TB burden experienced by HIV-infected children in TB endemic areas. Children with TB were significantly more immunocompromised and malnourished than those without TB, which may relate to the severity of the underlying HIV disease and/or reflect the detrimental effects of the TB itself. Most children were only commenced on HAART once they had advanced HIV disease (83% had WHO advanced or severe immune suppression), reflecting the large number of children who had been awaiting access to HAART, as well as conservative eligibility criteria. The high proportion of children with previous TB, among those not diagnosed with TB during the study period, can be explained by their relatively high mean age (34 months) compared to those who were diagnosed with TB (mean age 19 months). In this TB-endemic setting it is expected that older children would be significantly more likely to have had a previous episode of TB.

Most TB episodes were diagnosed in the 9 months prior to HAART initiation, during which time a diagnosis of TB is actively excluded. The number of TB diagnoses made pre-HAART was five times greater than post-HAART, which is even greater than the three fold reduction reported by Martinson *et al.*[28]. When calculating the number of TB cases per 100 patient years there was an eight fold reduction in TB episodes (53.3 versus 6.4), comparing the 9 months pre-HAART period to the post-HAART period. This was not a manifestation of overzealous diagnosis in the pre-HAART period, as the striking observation was even more pronounced when only bacteriologically confirmed cases were taken into account (18.3 versus 1.8). The reduction achieved is similar to that recorded with the use of universal INH preventive therapy [35]. It must be pointed out that the number of TB cases among those not on HAART may have been inflated by the fact that TB was frequently the presenting disease that lead to the diagnosis of HIV-infection and HAART initiation, especially before the institution of effective PMTCT programs and routine testing of HIV-exposed babies.

The clinical presentation of TB in our cohort was similar to what has previously been described [5,7-10] The presence or absence of a TB index case was recorded in less than 80% of children. This is an important oversight as careful scrutiny of the index case often provides important clues to identify drug-resistant disease in child contacts. Another interesting observation, although numbers were very small, is that the majority of index cases with documented sputum smear results were sputum smear-negative. Adults with sputum smear-negative pulmonary TB do transmit disease, albeit at a reduced rate. Current WHO guidelines do not recognize the potential transmission risk posed by exposure to a sputum smear-negative index case, especially if this is the child's mother or primary caregiver [36]. Weight loss or failure to thrive was the most frequent presenting symptom, but this may be caused by advanced HIV disease itself or by other HIV-related opportunistic infections. In addition accurate symptom definition, which is essential for optimal specificity [37], is impossible with retrospective analysis of routine clinical data.

The disease spectrum observed is comparable to that reported in HIV-uninfected children from the same area, although the number of children with disseminated (miliary) disease was significantly increased [9/134 (6.7%) vs 11/414(2.7%); OR 2.6, 95% CI 1.0–7.0] [38]. The timing of HAART introduction was largely dependent on the child's clinical condition; being introduced earlier in children with severe immune suppression. IRIS was not prominent in this group; in fact, disease progression in the absence of HAART often prompted its early initiation. The incidence of TB IRIS (7,4%) is slightly higher that that reported by Puthanakit *et al.*[30] and was higher among those with bacteriologically confirmed TB (4/46; 8.7%), compared to those without bacteriologically confirmed TB (6/88; 6.8%).

This study documents the frequency with which the management of TB in HIV-infected children deviates from standard protocol. Despite local [39] and international guidelines [40], therapy often has to be individualized. In this cohort, only 75/137 (55%) of TB episodes were treated with the standard 3-drug TB regimen; 33/137 (24%) for the standard 6 months. The proportion of children who received expanded treatment regimens was greater than that expected from the number of extra-thoracic and/or drug-resistant cases, which reflects extensive disease, high levels of co-morbidity and caution among clinicians who treat HIV-infected children with TB.

Adverse events that were probably drug-related occurred in only 7 children of whom 3 were on concurrent HAART. Although the absolute numbers are small it indicates that more than 5% of children may have experienced drug-related adverse events, with a further 4% experiencing non-drug-related adverse events. In total, adverse events necessitated treatment modification in nearly 10% of cases. This is important, as second line TB drugs are not as effective as first-line regimens, are more often associated with drug-related adverse events and result in longer treatment durations, with a concomitant increased risk of drug interactions and poor adherence. Although HAART was only suspended in a single child, stopping HAART for any period of time may impact negatively on the long-term prognosis of HIV-infected children.

The cohort in general did well, with 71% reporting positive outcomes. TB diagnosed after HAART initiation was associated with excellent outcomes. Although the reduced mortality recorded in children on HAART, compared to those not on HAART, at the time of TB diagnosis failed to reach statistical significance, no child who had been on HAART for more than 6 months prior to the TB episode died. Among the children who died, the majority had advanced HIV disease, and most died of HIV-related conditions other than TB. It is therefore not surprising that the group where HAART was introduced within 2 months of TB treatment had the worst outcomes, as the timing of HAART introduction was determined by the severity of the underlying HIV disease (clinical and immunological stage of the patient). These were young, severely immune suppressed and critically ill children in whom HAART was often given as a last resort. In this respect it is important to note that despite their grave clinical condition, 65% of these children survived with positive outcomes.

The early introduction of HAART was rarely (<10% of cases) associated with drug related adverse events or significant immune reconstitution phenomena. Two cases of BCG adenitis developed shortly after HAART initiation and probably represent BCG IRIS disease [41]. Although IRIS may have contributed to 4 deaths this remains highly speculative, as 2 deaths occurred relatively late (more than 5 months after HAART initiation) and the other 2 had progressive MDR TB; the progressive and drug-resistant nature of their disease was probably responsible for their demise.

Being retrospective, our study is limited by incomplete data and missing records. However, we were able to retrieve essential data in the vast majority of children (>95%) and believe that the information provided allows accurate assessment of the diagnostic challenges related to TB and HIV in everyday clinical practice. Although this is a hospital-recruited cohort, significant selection bias is unlikely as, during the study period, TCH functioned as the only point of access to HAART for children from the surrounding areas.

## Conclusion

The study confirms the high incidence of TB among HIV-infected children in TB endemic settings. Although the management of TB in HIV-infected children may be complicated, the majority of children achieved a positive outcome. HAART initiation was followed by a substantial reduction in the incidence of TB, yet children only accessed HAART at an advanced stage of HIV disease. For HIV-infected children living in TB endemic areas, starting HAART at an earlier stage is likely to reduce TB-related morbidity and mortality.

## Competing interests

The author(s) declare that they have no competing interests.

## Authors' contributions

EW was the principal investigator who contributed to the conception and design of the study, collected, entered and analyzed the data, and assisted with drafting the manuscript. MFC contributed to the conception and design of the study, data interpretation and drafting the manuscript. HR contributed to the conception and design of the study, data interpretation and drafting the manuscript. HSS contributed to the conception and design of the study, read all the chest radiographs, and assisted with drafting the manuscript. LOW constructed the database, assisted with data analysis and interpretation, and with drafting the manuscript. BJM acted as the study supervisor, assisted with the conception and design of the study, data analysis and interpretation, drafted the manuscript and acted as corresponding author. All authors read and approved the final manuscript.

## Pre-publication history

The pre-publication history for this paper can be accessed here:


